# UpToDate versus DynaMed: a cross-sectional study comparing the speed and accuracy of two point-of-care information tools 

**DOI:** 10.5195/jmla.2021.1176

**Published:** 2021-07-01

**Authors:** Glyneva Bradley-Ridout, Erica Nekolaichuk, Trevor Jamieson, Claire Jones, Natalie Morson, Rita Chuang, Elena Springall

**Affiliations:** 1 glyneva.bradley.ridout@utoronto.ca, Gerstein Science Information Center, University of Toronto Libraries, University of Toronto, Canada; 2 erica.lenton@utoronto.ca, Gerstein Science Information Center, University of Toronto Libraries, University of Toronto, Canada; 3 trevorjam@me.com, Department of Medicine, University of Toronto, Division of General Internal Medicine, Unity Health Toronto, Canada; 4 claire.jones@sinaihealth.ca, Department of Obstetrics and Gynaecology, University of Toronto, Mount Sinai Fertility, Sinai Health System, Toronto, Canada; 5 natalie.morson@sinaihealth.ca, Department of Family and Community Medicine, University of Toronto, Mount Sinai Academic Family Health Team, Sinai Health System, Toronto, Canada; 6 rita.chuang@mail.utoronto.ca, Department of Obstetrics and Gynaecology, University of Toronto, Canada; 7 elena.springall@utoronto.ca, Gerstein Science Information Center, University of Toronto Libraries, University of Toronto, Canada

**Keywords:** point of care tools, UpToDate, DynaMed

## Abstract

**Objective:**

To compare the accuracy, time to answer, user confidence, and user satisfaction between UpToDate and DynaMed (formerly DynaMed Plus), which are two popular point-of-care information tools.

**Methods:**

A crossover study was conducted with medical residents in obstetrics and gynecology and family medicine at the University of Toronto in order to compare the speed and accuracy with which they retrieved answers to clinical questions using UpToDate and DynaMed. Experiments took place between February 2017 and December 2019. Following a short tutorial on how to use each tool and completion of a background survey, participants attempted to find answers to two clinical questions in each tool. Time to answer each question, the chosen answer, confidence score, and satisfaction score were recorded for each clinical question.

**Results:**

A total of 57 residents took part in the experiment, including 32 from family medicine and 25 from obstetrics and gynecology. Accuracy in clinical answers was equal between UpToDate (average 1.35 out of 2) and DynaMed (average 1.36 out of 2). However, time to answer was 2.5 minutes faster in UpToDate compared to DynaMed. Participants were also more confident and satisfied with their answers in UpToDate compared to DynaMed.

**Conclusions:**

Despite a preference for UpToDate and a higher confidence in responses, the accuracy of clinical answers in UpToDate was equal to those in DynaMed. Previous exposure to UpToDate likely played a major role in participants' preferences. More research in this area is recommended.

## INTRODUCTION

Point-of-care information (POCI) tools are online information resources that provide evidence-based, distilled information designed to help clinicians find answers quickly to their clinical questions. Two major POCI tools on the market today are DynaMed and UpToDate.

It is generally understood that use of POCI tools is necessary for finding timely answers to questions [1]. They are also associated with increased ability to answer clinical questions and improved patient care [2, 3, 4]. However, it is less clear which POCI tool provides the most efficient, effective, and easy path to answers in a clinical setting. UpToDate is a very popular and effective tool, often cited as the preferred resource for clinical questions among hospitalists in a variety of settings [5, 6]. A recent survey of user preferences between two POCI tools, UpToDate and ClinicalKey, indicated a preference for UpToDate in the clinical setting [7]; however, the study authors concluded that ClinicalKey was more suitable as a didactic or classroom tool. A crossover randomized controlled trial that was conducted in 2011 compared the speed and accuracy of UpToDate with PubMed Clinical Queries and concluded that using UpToDate resulted in higher proportions of accurate answers in less time [8]. However, no previous study has compared UpToDate with a POCI tool similar in purpose, functionality, breadth of content, currency, and quality of evidence.

DynaMed is a relatively new tool and is promoted as an appropriate alternative to UpToDate. Given increasing subscription costs for these resources, librarians in both academic and hospital settings need to make informed choices when purchasing POCI tools and engage users in decision-making [9]. It is also important to introduce different clinical tools to physicians to broaden their comfort level in using different software in an ever-changing clinical and technological world.

Based on their specifications, UpToDate and DynaMed appear to serve the same purpose and are of the same overall quality; however, there are major differences in their interface, search functionality, and presentation of information. There are also differences in conflict of interest between the two tools. A 2014 study examining six articles in both UpToDate and DynaMed determined that all UpToDate articles had at least one conflict of interest, whereas DynaMed articles had none [10]. While several evaluation studies of POCI tools have been published [11, 12], there is currently no experimental evidence published comparing the ease-of-use, effectiveness, or efficiency between these two tools. Anecdotal evidence we have gathered to determine user preference for DynaMed and UpToDate has been inconclusive.

This study was designed to meet this gap and inform collections decisions made by health sciences librarians and to inform the choice of resources that physicians subscribe to individually and use at the point of care. We conducted an experiment with fifty-seven residents in obstetrics and gynecology (OBGYN) and family medicine comparing the accuracy, speed, user confidence, and satisfaction in clinical answers retrieved using UpToDate and DynaMed.

## METHODS

### Participants and settings

Approval was obtained from the University of Toronto Health Sciences Research Ethics Board in September 2017. Our target population for this study was OBGYN and family medicine residents at the University of Toronto in any year of residency. Recruitment took place via emails sent to the targeted resident groups inviting participation in the study (Appendix 1). The study took place with variously sized groups of participants between February 2017 and December 2019 at three hospital sites in Toronto: St. Michael's Hospital of Unity Health Toronto, Toronto General Hospital of the University Health Network, and Women's College Hospital.

Participation in this study was voluntary. Prior to each iteration of the experiment, an investigator verbally explained the study, and written informed consent was obtained. Participants were offered a $10 Starbucks gift card to participate. Participants were able to withdraw from the study at any point with no consequence.

## INTERVENTIONS

Each study group began with separate five-minute tutorials on UpToDate and DynaMed, which were delivered by the librarian investigators. The tutorials covered a brief overview of how to conduct a search in each tool. Features such as levels of evidence scales and searching within a topic summary were demonstrated. The content and features covered in the UpToDate and DynaMed tutorials were similar, including the same search topic used in the example search demonstration. The tutorials were designed to give participants exposure to each tool prior to the experiment and focused on specific characteristics of each tool.

The experiment required that participants locate answers using the mobile versions of the POCIs, so each participant was invited to download the apps onto their personal mobile devices. Backup mobile devices with the apps pre-installed were available if a participant preferred not to download the applications on their own device or if there were any technical issues in doing so.

**Figure 1:**
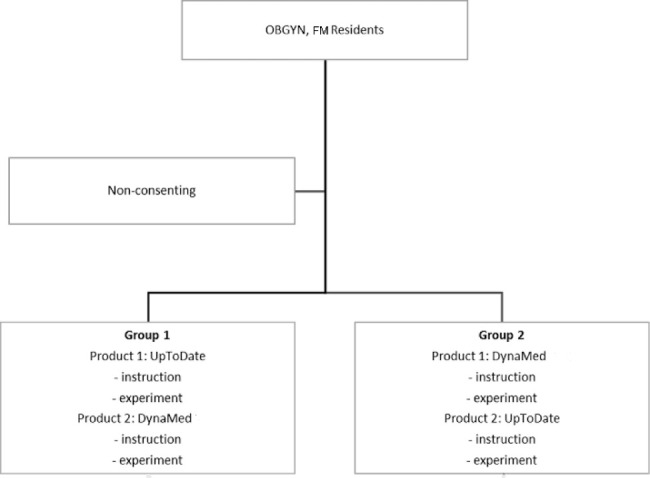
Participant pathways

The study involved each participant answering two clinical questions in one POCI followed by two different questions in the other POCI. The clinical questions were drawn from *PROLOG Obstetrics* [13] and *PROLOG Gynecology and Surgery* [14] for OBGYN residents. Family medicine residents received questions about scenarios drawn from the above two sources as well as the MKSAP 18: medical self-assessment program [15]. A total of four clinical questions per participant were randomly assigned from seven questions per specialty. It was verified that answers to each question could be located in both UpToDate and DynaMed. Randomization took place using the random integer set generator from Random.org [16]. Following randomization, a set of experiment packages were created for each specialty that contained a background survey and the four randomly generated clinical questions. A coin toss took place to determine which packages would begin the experiment with DynaMed and which would begin the experiment with UpToDate (Figure 1). The packages were then sealed until they were opened by each participant during the study.

### Measurements

The primary outcome measures of the study were time to answer and answer accuracy. The secondary outcome measures were confidence in using the tool and satisfaction with the tool.

A background survey collected baseline characteristics of each participant including specialty, postgraduate year of training (PGY#), and age (Appendix 2). Participants were also asked questions about their familiarity, comfort level, and preferences with specific POCI tools in answering clinical questions. An open-ended question was used to ask each participant to state their preferred source of clinical information in general.

Following completion of the background survey, participants began to answer their four randomly generated clinical scenarios. An example of clinical scenarios and questions asked can be found in Appendix 3. The first two questions were answered using their randomly allocated start POCI tool, and the second two questions were answered using the other POCI tool. To measure time to answer, each participant self-recorded their start and end time (up to a maximum of ten minutes) in the space provided on the recording sheet for each clinical scenario.

To measure accuracy, participants completed multiple-choice questions or recorded that they did not find an answer. Measures of confidence and satisfaction for each tool were recorded using Likert scales following each clinical question. When participants completed the background survey and all four clinical questions, all materials were returned to the investigators, and the experiment concluded.

### Statistical analysis

We did not review any participant results until the conclusion of all data collection. Data from the background survey and clinical questions were entered into Microsoft Excel by a graduate student assistant who was not otherwise involved in the investigation. Two researchers reviewed the Excel data to ensure accuracy.

For answer accuracy, each participant was given a score of 0 or 1 for each question (0 being an incorrect answer, and 1 being correct). As each participant answered two questions in each POCI tool, the total accuracy of each tool was therefore measured out of 2. For time to answer, each participant's time to answer each clinical question was converted into seconds. The time to answer both questions was combined to indicate the total time for each tool. Statistical analysis took place in Stata [17]. Accuracy, confidence, and satisfaction were compared using Wilcoxon Signed Rank tests, and time to answer was analyzed using a paired t-test.

## RESULTS

### Characteristics of participants

A total of 57 residents took part in the experiment, and all fully completed the study; 56% (n=32) were family medicine residents, and 44% (n=25) were OBGYN residents. The majority of residents were in their first year of residency, and the average age of participants was 27 years. In the open-ended question where participants were asked to state their preferred source of clinical information in general, 10 unique information sources were written by participants, which were tallied to report the total number of mentions. Table 1 displays the full demographic information of the participants.

**Table 1:** Baseline demographic characteristics of participants

Characteristic	All participants (n=57)
Age—mean (SD)	28 (2.7)
Specialty—no. (%)	
Family medicine	32 (56)
Obstetrics and gynecology	25 (44)
Postgraduate year—no. (%)	
PGY1	39 (68)
PGY2	11 (19)
PGY3	5 (9)
PGY4	1 (2)
PGY5	1 (2)
Preferred information source—no. of mentions	
UpToDate	54
Guidelines	14
Articles	3
Personal notes	2
Medscape	1
PubMed	1
Google	1
Faculty/peers	1
DynaMed	1
BMJ	1

Nearly all (98%, n=56) participants had used UpToDate prior to the experiment, and the remaining one participant selected that they were unsure. Those who had used UpToDate previously reported using it for an average of 3.9 years. Every participant stated that they would recommend UpToDate to a peer in the future. In comparison, 19% (n=11) of participants had used DynaMed prior to the experiment, and 79% (n=45) never had. One participant was unsure. Those who used DynaMed previously reported using it for an average of 2.5 years. Three participants stated that they would recommend DynaMed to a peer, and the remaining eight selected “maybe” or “no.” Participants were also asked to rate their comfort using each tool on a Likert scale (Table 2).

**Table 2:** Baseline information for participant experience and use of POCI tools

Characteristic	UpToDate	DynaMed
Prior Experience—no. (%)		
Yes	56 (98)	11 (19)
No	0 (0)	45 (79)
Unsure	1 (2)	1 (2)
Years of use*—mean (SD)	3.9 (1.6)	2.5 (2.0)
Self-declared comfort level on Likert scale*—no. (%)		
Very comfortable	39 (70)	1 (9)
Somewhat comfortable	17 (30)	2 (18)
Neither	0 (0)	4 (36)
Somewhat uncomfortable	0 (0)	3 (27)
Very uncomfortable	0 (0)	1 (9)
Recommendation*—no. (%)		
Yes	56 (100)	3 (27)
No	0 (0)	3 (27)
Maybe	0 (0)	5 (45)

* Only considers subset of population with prior experience using POCI tool

### Accuracy

Participants' clinical answers using UpToDate and DynaMed were equally accurate. The mean number of questions answered correctly by each participant was 1.35 when using UpToDate and 1.36 when using DynaMed, which was not a statistically significant difference (Wilcoxon Signed Rank test, z=−0.253; obs=57; *p*=0.80).

### Time to answer

The total time to answer was on average 155 seconds (i.e., over 2.5 minutes) faster using UpToDate compared to DynaMed. The mean time to find an answer was 304 seconds for UpToDate and 459 seconds for DynaMed, which was a statistically significant difference (paired t-test, t=−3.58; df=56; *p*=0.0007).

### Satisfaction and confidence

Participants were more confident in the answers they found in UpToDate compared to DynaMed. The mean confidence score was 6.8 out of 10 for UpToDate and 5.8 out of 10 for DynaMed, which was a statistically significant difference (Wilcoxon Signed Rank test, z=2.985; obs=57; *p*=0.0028).

Participants were also overall more satisfied with UpToDate compared to DynaMed. The mean satisfaction score was 7.9 out of 10 for UpToDate and 6 out of 10 for DynaMed, which was a statistically significant difference (Wilcoxon Signed Rank test, z=4.78; obs=57; *p*< 0.0001).

## DISCUSSION

Our confidence and satisfaction score findings align with previous research indicating that UpToDate is clinicians' preferred POCI [7]. However, to our knowledge, ours is the first study to demonstrate that despite a preference for UpToDate and a higher confidence in responses, the accuracy of clinical answers obtained using UpToDate was equal to that using DynaMed.

While there was no significant difference in the accuracy of answers retrieved from the two POCI tools, the 2.5-minute difference in time to answer retrieval is statistically significant and arguably impactful to clinical practice, considering the number of clinical questions that residents could encounter during an on-call shift [3, 18].

There are likely strong links among participants' time to answer, confidence, and satisfaction with each POCI tool and their existing exposure prior to the experiment. Out of 57 participants, 54 stated UpToDate as their preferred source to find clinical information, and only 1 participant mentioned DynaMed. Further, 98% (n=56) of participants had used UpToDate at some point before the experiment, compared to only 19% (n=11) of participants who had previously used DynaMed. It is unlikely that the short 5-minute training session provided on each tool would have corrected for this difference in exposure. This point is relevant for generalizability of these study findings to other physicians who have previous experience with one or both of these POCI tools.

It is also notable to consider the years of experience that the participants had with each tool before the experiment. Those who had used UpToDate before the experiment had been using it for an average of 3.9 years, whereas those who had used DynaMed in the past had been using it for an average of 2.5 years. Therefore, existing familiarity and years of experience with UpToDate may have played a role in how quickly the participants were able to find answers in UpToDate compared to DynaMed. These items align with the fact that UpToDate has been on the market as a POCI tool longer than DynaMed.

There are some limitations of this study to acknowledge. Firstly, experiment groups ranged in size from one to twenty participants at once, and participant groups that were smaller may have had a more personalized experience during the instructional tutorial. Our survey instrument was proofread by all investigators, including clinicians, but was not tested on members of the study population. Our participants ranged in their professional careers from PGY1 to PGY4, which may have resulted in a range of experience and knowledge coming into the experiment. Finally, measuring time to answer by having participants keep track and record their own time may have resulted in some inaccurate reporting.

This study can be used to encourage additional research on this topic. In particular, we believe that the previous experience our participants had using UpToDate may have had a major impact on our results. A suggestion for future research would be to conduct a similar study with students early in medical school, before they have had as much exposure to POCI tools. This would help to validate whether previous experience plays a significant role in time to answer, confidence, and satisfaction with UpToDate versus DynaMed.

In conclusion, we found that OBGYN and family medicine residents were equally successful in finding accurate answers to clinical questions using UpToDate and DynaMed. Time to answer, confidence in, and satisfaction with those answers was significantly better with UpToDate, but this was likely influenced by baseline user experiences with the two tools. The equivalent accuracy between tools justifies more deliberate and early exposure to both tools in training so that future assessments can determine whether the efficiency to find an answer is purely due to prior experience or an inherent function of the tools themselves.

Overall, this study is relevant for physicians in all areas of medicine who need to be able to look up reliable clinical information in a timely fashion for providing patient care. This study should reassure physicians that both POCI tools demonstrate equivalent accuracy for answering clinical questions in a short time frame with a slightly reduced time using UpToDate. This study is also relevant for health sciences librarians to consider collections decisions and education around these two POCI tools.

## Data Availability

Data associated with this article (including the Research Ethics Board application, tutorials, example participant package, recruitment email, and study consent form) are available in the Open Science Framework at https://osf.io/dkvsg/?view_only=bbe19d40f0584f44830920e234fea1b2.

## SUPPLEMENTAL FILES

**Appendix 1:** Example recruitment email

**Appendix 2:** Background survey

**Appendix 3:** Example clinical scenarios
