# Supplementary material for: UpToDate versus DynaMed: a cross-sectional study comparing the speed and accuracy of two point-of-care information tools
Source: J Med Libr Assoc. 2021 Jul 1;109(3):382–7. doi: 10.5195/jmla.2021.1176 (PMC8485969; doi:10.5195/jmla.2021.1176)
Supplement: Supplementary file 1 — Appendix 1: Example recruitment email [file jmla-109-3-382-s01.docx]

Appendix 1: Example Recruitment Email

**Subject Line:** Opportunity to Participate in Research: Compare UpToDate versus DynaMed Plus and receive a Starbucks Gift Card!

***

You are invited to participate in a study titled:

**UpToDate versus DynaMed Plus: A mixed methods study comparing the speed and accuracy of two point-of-care information resources in answering clinical questions.**

*Participation in the study will be voluntary and will occur during your own personal time*.

The goal of this study is to determine whether two point-of-care information tools, UpToDate and DynaMed Plus, are equivalent in terms of accuracy and efficiency.

We are recruiting residents in Obstetrics and Gynaecology or Family Medicine at the [name of institution] to participate in a study to evaluate both resources’ ability to find the answers to clinical questions. You will be asked to use the resource to which you have been assigned to answer 2 clinical questions in 10 minutes each and will evaluate the utility of the clinical resource in providing the necessary information for each particular question. Following that, you will be asked to use the other resource to evaluate 2 more clinical questions within 10 minutes. Participation in this study requires filling out the a 5-minute baseline survey about yourself prior to starting the assessment. The whole study will take under 1 hour to complete. Following the study, you are invited to stay behind for a short focus group designed to understand your experience utilizing both resources.

Participation in the study will be confidential and will not affect your residency with [name of institution]**.** It will take approximately 1 hour to complete the study. You will be given a $10 gift card after you complete the study. If you elect to participate in the focus group, you will be given another $10 gift card**.** Information learned from this study may help other people use clinical resources in the future.

If you wish to participate in this study, please contact the research team using the information provided below. Should you choose to participate, your participation will enhance and facilitate this research project and the larger research mission of the Faculty.

We thank you for considering this request.
