# Supplementary material for: UpToDate versus DynaMed: a cross-sectional study comparing the speed and accuracy of two point-of-care information tools
Source: J Med Libr Assoc. 2021 Jul 1;109(3):382–7. doi: 10.5195/jmla.2021.1176 (PMC8485969; doi:10.5195/jmla.2021.1176)
Supplement: Supplementary file 2 — Appendix 2: Background survey [file jmla-109-3-382-s02.docx]

Appendix 2: Background Survey

1. What is your specialty: ________________________________________________

2. What PGY# are you: __________________________________________________

3. What is your age: ____________________________________________________

4. Do you have prior experience using UpToDate?

¨ Yes

¨ No

¨ Unsure

**If you answered YES to question 4, please answer the following questions.**

**If you answered NO or UNSURE, please skip to question 5.**

4a. How many years have you been using UpToDate? __________________________

4b. How comfortable are you using UpToDate?

5 Very comfortable

4 Somewhat comfortable

3 Neither comfortable nor uncomfortable

2 Somewhat uncomfortable

2 Very uncomfortable

4c. Would you recommend UpToDate to a peer?

¨ Yes

¨ No

¨ Maybe

4d. How do you use UpToDate most often:

¨ Website on a laptop, desktop computer

¨ Website on a phone (mobile website)

¨ UpToDate app

4e. How were you first introduced to UpToDate? Select all that apply:

¨ Librarian/Library website

¨ Peer/Friend

¨ Professor/Supervisor/Faculty member

¨ Can’t remember

¨ Other (explain): _________________________

5. Do you have prior experience using Dynamed Plus?

¨ Yes

¨ No

¨ Unsure

**If you answered YES to question 5, please answer the following questions.**

**If you answered NO or UNSURE, please skip to question 6.**

5a. How many years have you been using Dynamed Plus? __________________________

5b. How comfortable are you using Dynamed Plus?

¨ Very comfortable

¨ Somewhat comfortable

¨ Neither comfortable nor uncomfortable

¨ Somewhat uncomfortable

¨ Very uncomfortable

5c. Would you recommend Dynamed Plus to a peer?

¨ Yes

¨ No

¨ Maybe

5d. How do you use Dynamed Plus most often? Select one:

¨ Website on a laptop, desktop computer

¨ Website on a phone (mobile website)

¨ Dynamed Plus mobile app

5e. How were you first introduced to Dynamed Plus? Select all that apply:

¨ Librarian/Library website

¨ Peer/Friend

¨ Professor/Supervisor/Faculty member

¨ Can’t remember

¨ Other (explain): _________________________

6. On a scale of 1-4 where 1 is not confident at all and 4 is very confident, how confident are you in your ability to find information to support your clinical decisions?

4 Very confident

3 Somewhat confident

2 Somewhat not confident

1 Not confident at all

7. What is your preferred source of information to support your clinical decisions?
