# Supplementary material for: UpToDate versus DynaMed: a cross-sectional study comparing the speed and accuracy of two point-of-care information tools
Source: J Med Libr Assoc. 2021 Jul 1;109(3):382–7. doi: 10.5195/jmla.2021.1176 (PMC8485969; doi:10.5195/jmla.2021.1176)
Supplement: Supplementary file 3 — Appendix 3: Example clinical scenarios [file jmla-109-3-382-s03.docx]

Appendix 3: Example Clinical Scenarios

Example OBGYN Scenarios

Scenario 1

**Item 76 –**Severe rhesus disease in pregnancy

A 33-year-old woman, gravida 4, para 1, at 18 weeks of gestation presents for her first prenatal visit. She has a history of rhesus (Rh) alloimmunization with delivery of an anemic neonate that required exchange transfusion after birth in her most recent pregnancy. She reports certain paternity and that her husband is a known RhD homozygote. The most appropriate next step in management is to assess

1. amniotic fluid ∆OD₄₅₀
2. fetal hemoglobin level
3. fetal middle cerebral artery peak systolic velocity
4. fetal RhD antigen status
5. the maternal anti-D titer

Scenario 2

**Item 102 –**Seizure disorder medications

A 27-year-old woman at 7 weeks of gestation is on multiple medication to control her seizures. You counsel her that the antiepileptic drug with the highest teratogenic risk is

1. phenytoin
2. valproate
3. lamotrigine
4. levetiracetam
5. carbamazepine

Example Family Medicine Scenarios

Scenario 1

**Item 8 – GENERAL INTERNAL MEDICINE**

A 91-year-old woman with advanced dementia is examined in her extended-care facility for a routine evaluation. She is nonverbal, incontinent of urine and stool, largely bedbound, and dependent on others for all activities of daily living. The patient’s nurse notes that the patient has continued to lose weight despite being actively fed but raises no other concerns. Medical history is significant for hypertension, and her only medication is amlodipine.  
On physical examination, blood pressure is 132/87 mm Hg; other vital signs are normal. The patient appears cachectic with temporal wasting. She is awake but is unresponsive to questions. Mucous membranes are moist. She does not appear to have any pain. There are mild early contractures of her ankles and hips. The remainder of the examination, including skin examination, is unremarkable.

**Which of the following is the most appropriate intervention for preventing pressure ulcers in this patient?**

1. Alternating-air mattress
2. Enteral nutrition
3. Foam mattress overlay
4. Frequent repositioning

Scenario 2

**Item 89 –**Multiple sclerosis

A 34-year-old woman with a history of relapsing and remitting multiple sclerosis sees you for a preconception consultation. She asks about the change of having a relapse during pregnancy. You inform her that her chance of relapse is highest during the

1. first trimester
2. second trimester
3. third trimester
4. postpartum period
